# Comparison of Interface Pressures and Subjective Comfort of Pressure-Relieving Overlays on the Operating Table for Healthy Volunteers

**DOI:** 10.3390/ijerph18052640

**Published:** 2021-03-05

**Authors:** Min Jung Han, Sangjin Ko

**Affiliations:** 1 Kyungpook National University Hospital, Daegu 41944, Korea; rngemini@naver.com; 2 Department of Nursing, University of Ulsan, Ulsan 44610, Korea

**Keywords:** pressure ulcer, operating tables, perioperative nursing, pressure, sacrum, heel

## Abstract

(1) Background: Pressure ulcers in the hospital setting occurring within 72 h after surgery are called perioperative pressure injuries. The aim of this study was to provide data for the prevention of perioperative pressure injuries following the use of pressure-relieving overlays by measuring the interface pressures and subjective comfort. (2) Methods: This study is based on a repeated measures design. The subjects included 30 healthy volunteers aged 18 to 57 years. Interface pressures of the sacrum and both heels were measured in the supine position, and the subjective comfort was evaluated with visual analog scale after applying polyurethane foam, gel pad, and egg crate foam for relief. (3) Results: The pressures in the sacrum and both heels were the lowest with polyurethane foam, and the subjective comfort was the highest. (4) Conclusions: Inexpensive polyurethane foam with satisfactory pressure relief is recommended as an overlay for surgical patients.

## 1. Introduction

The purpose of this study is to provide data for the prevention of pressure injury following the use of inexpensive disposable and/or repeatable pressure-relieving overlays by measuring the interface pressures and subjective comfort on the sacrum and heels.

Pressure injuries (referred to as pressure ulcers) are chronic or repetitive pressure on the bony prominence resulting in local damage or necrosis of skin tissue [1]. In particular, a hospital-acquired pressure injury causes pain and disability and increases morbidity due to other diseases [2]. Consequently, these complications increase the duration of hospital stay, which can even lead to death [2]. Although all patients admitted to hospitals face the potential risk of pressure injury, the occurrence of such injuries in hospitals can also be used as an indicator of their quality because they can be prevented to some extent [2,3].

Pressure injuries in hospitals occurring within 72 h after surgery are called perioperative pressure injuries [4]. It has been reported to the incidence of perioperative pressure injuries ranges from 0.3 to 57.4% [5], and some studies have reported up to 69% [6]. Because most major surgeries performed under general or spinal anesthesia render patients unconscious and immobile, constant pressure is applied to the body tissues that are in contact with the surgical bed [7]. Eventually, this sustained compression causes pressure injuries, especially inducing muscle breakdown [8]. The time of prolonged pressure and shear forces are important factors in causing the pressure injuries [9], but it is impossible to reduce the operation time or change the position during surgery to prevent the occurrence. Therefore, a variety of pressure-relieving overlays, such as an alternating pressure overlay [10,11] or dressings [9], are used to reduce the pressure and shear force during surgery. The use of these materials during surgery is effective and is known to decrease the incidence of pressure bedsores by 0.51% [12].

However, sometimes hospitals face limitations in using high-tech supplies for patient care due to the high cost. Especially in the National Health Insurance System in South Korea, the hospital first treats the patient, and the Health Insurance Review & Assessment Service of the government evaluates and compensates the cost required only for essential and minimal treatment [13]. Korean hospitals should search the cost-effective products that can be reused for a long time, as pressure-relieving overlays cannot be compensated by the Health Insurance Review & Assessment Service. However, few studies have suggested the quantitative pressure level on specific sites according to the use of pressure-relieving overlays in operating room of South Korea.

Therefore, the purpose of this study is to provide data for the prevention of pressure injury following the use of inexpensive disposable and/or repeatable pressure-relieving overlays by measuring the interface pressures and subjective comfort on the sacrum and heels.

## 2. Materials and Methods

### 2.1. Study Design

This study is based on a repeated measures design to compare the pressure on the sacrum and heels and evaluate subjective comfort after applying pressure-relieving overlays, including polyurethane (PU) foam, gel pads, and egg crate (EC) foam, which are routinely used to prevent postoperative pressure ulcers in operating rooms in South Korea.

### 2.2. Sample and Setting

The study subjects were 30 healthy volunteers aged 24 to 57 years. The calculated sample size was 28 for one-way analysis of variance (ANOVA) using the G*Power 3.1 program [14] (significance level: 0.05; power: 95%; effect size: 0.40; group number: 4). A total of 30 subjects were selected for the study, considering the dropout rate of 10%.

### 2.3. Measures

#### 2.3.1. Interface Pressure

**Figure 1:**
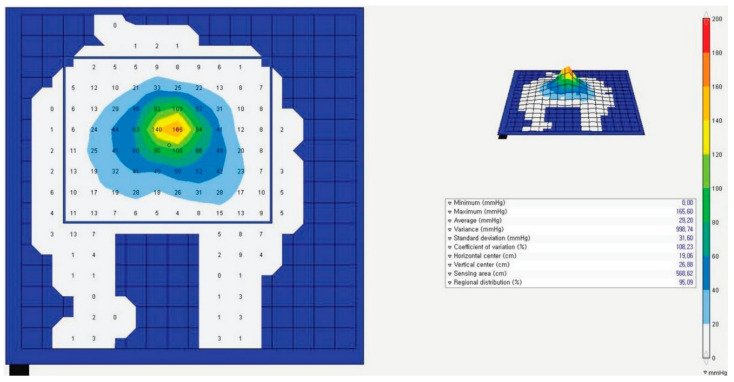
Sample of the pressure-distribution-analyzing screen.

Interface pressure is defined as the pressure at the junction between the skin and the support surface. In this study, it was measured using the Force Sensitive Application (FSA) pressure mapping system (VISTA Medical, Winnipeg, MB, Canada), which is connected to computer and a module. It is composed of a flexible pressure sensing mat (238.0 × 190.4 × 0.2 mm) and contains 256 (16 × 16) sensors with a diameter of 23.8 × 23.8 mm. It can measure pressure up to 200 mmHg (Figure 1).

#### 2.3.2. Subjective Comfort

The subjective comfort of lying on the operating bed was measured with a visual analog scale (VAS) based on values ranging between 0 (very uncomfortable) and 10 (very comfortable) immediately after pressure measurement.

### 2.4. Data Collection and Analysis

The general characteristics of all subjects, including gender, age, height, and weight, were evaluated. They wore a surgical gown similar to that used by surgical patients and lay in a supine position on the surgical bed. The interface pressure was measured on the standard mattress in the operating room without any pressure-relieving overlays and then on the surface of the standard mattress using a PU foam (25 mm thickness), gel pad (13 mm thickness), and EC foam (25 mm thickness). The pressure-sensing mat was placed on the hip and two feet and was measured for 10 min by repeating every 10 s. The distribution of pressures was analyzed to the isobar and contour using FSA 4.0 software (VISTA Medical, Winnipeg, MB, Canada). The pressure values were extracted from the center on the screen of the 10 × 8 pressure sensor. To measure the subjective, the subject response was assessed immediately using VAS.

The collected data were analyzed using the IBM SPSS Statistics for Windows, Version 22.0 (IBM corp, Armonk, NY, USA). The general characteristics of the subjects are presented as real numbers, percentages, averages, and standard deviations. In order to compare the interface pressure and subjective comfort by the pressure-relieving overlays, the averages were compared using one-way ANOVA and Scheffe post-test.

### 2.5. Ethical Approval

All research procedures were conducted after approving from the Institutional Review Board of K University Hospital (No: 2015-11-004). All subjects were fully informed regarding the purpose and necessity of the study and the possibility of withdrawal of their participation arbitrarily at any time. Written informed consent was obtained, and all subjects were provided with a gift as compensation for participation in the study.

## 3. Results

### 3.1. General Characteristics of Participants

There was more female (93.5%) than male (6.5%), and the average age of the participants was 38.42 years. The average height of the subjects was 161.06 cm, and the weight was 57.32 kg. The body mass index (BMI) was 12.9% for underweight subjects, 64.5% for normal subjects, and 9.7% for overweight subjects (Table 1).

### 3.2. Comparison of the Average Interface Pressure on the Sacrum and Both Heels

The mean interface pressure of the sacrum was higher in the order of gel pad (57.42 ± 35.61), standard mattress (56.10 ± 23.03 mmHg), EC foam (47.03 ± 32.29), and PU foam (31.55 ± 13.60 mmHg), and there was a significant difference (F = 5.82, *p* = 0.001). The post-test results show that the interface pressure for the PU foam was significantly lower than that of the general mattress and gel pad.

The mean interface pressure of both heels was higher in the order of standard mattress (84.68 ± 36.47 mmHg), gel pad (63.58 ± 32.31 mmHg), EC foam (48.10 ± 27.01 mmHg), and PU foam (41.39 ± 13.29 mmHg), and there was a significant difference (F = 13.97, *p* < 0.001). The post-test results show that the interface pressure for the standard mattress was significantly higher than that of the other materials, and that of the PU foam was significantly lower than that of the standard mattress and gel pad (Table 2).

### 3.3. Comparison of Subjective Comfort

The subjective comfort measured by VAS was higher in the order of PU foam (7.00 ± 2.09), EC foam (6.57 ± 2.01), gel pad (5.91 ± 1.74), and standard mattress (5.38 ± 1.76), and there was a significant difference (F = 13.46, *p* < 0.001). Among them, the comfort of the EU foam was significantly higher than that of the standard mattress and gel pad, and the comfort of the PU foam was significantly higher than that of the standard mattress (Table 3).

## 4. Discussion

Hospital-acquired pressure injuries increase the time and cost of care. Factors that predict an individual’s pressure injury can be divided into internal (such as nutritional status) [15] and external (such as pressure, shear force, time, temperature, and humidity) factors [16,17]. Since most patients who are hospitalized and undergoing surgery have poor intrinsic factors, it is best to control the external environment in the operating room to prevent perioperative pressure injuries. In a systematic review [18], to prevent pressure injury, adequate nutritional status, use of moisturizers, and mattress overlays are suggested as interventions to prevent pressure injury. Therefore, the use of an appropriate pressure-relieving overlay is an effective method for preventing pressure injuries.

The results of this study show that the average interface pressure between the sacrum and both heels was the lowest in PU foam and the highest in the standard mattress. Among them, the interface pressure with PU foam was significantly lower than that of the standard mattress and gel pads, which is consistent with the results of the study conducted by Keller [19], who investigated pressures according to four types of mattresses in two positions. In this study, the average interface of the gel pad was the highest in the sacrum (57.42 ± 5.61) and the second highest in both heels (63.58 ± 32.31). This result supports previous studies whose results show that the incidence of pressure ulcers is higher when using a gel pad (34.3%) than when using a PU foam (16.7%) [20], and that PU foam has an effect on pressure distribution [21] but gel pads do not [22]. However, according to the survey of the supporting surface used in Korea [23], 57% of nurses in the operating room thought that the gel type was the most effective. Therefore, there is a need for education to improve the awareness of nurses regarding the role of overlays in the prevention of pressure injury in operating rooms in South Korea. Furthermore, usually, patients report a higher surface pressure than healthy people [24]. In this study, the mean interface pressure of the sacrum was 56.10 ± 23.03 mmHg, and this was lower than that of 10 patients (105.7 ± 22.4) in the study conducted by Duetzmann et al. [24]. Therefore, considering that the subjects of this study were healthy people, the interface pressure on patients would be higher, and this suggests that use of appropriate pressure-relieving overlays is needed to prevent pressure injuries.

Based on the measurement of subjective comfort, PU foam was significantly higher than the standard mattress. While this cannot be compared in the absence of a previous study investigating subjective comfort, the use of PU foam may cause less discomfort than undergoing local or spinal surgery on a standard mattress. However, the gel pad was less comfortable than the standard mattress. Interestingly, in this study, higher subjective comfort was associated with lower interface pressure. PU foam with the lowest average interface pressure showed the highest subjective comfort. Therefore, it is also worth considering the provision of comfort according to the pressure-relieving overlay, unless the surgery requires reposition after anesthesia. However, this needs further study.

In the study conducted by Romanelli et al. [25], when the supine position was assumed on a regular mattress, the total average pressure was 30–40 mmHg, and the pressure at the bone elevation was 70–100 mmHg. At this pressure, the pathological changes in the tissue appeared within 2 h, so position change was required every 2 h. However, it is impossible to change the position this frequently, and if a surgery time of more than 2 h is expected, it is essential to measure the appropriate interface pressure and use a pressure-relieving overlay as needed. In fact, the 2 h operation is much longer in reality because it involves pre-operative and recovery periods. Recently, effective pressure-relieving overlays, such as alternating pressure [10,11] and viscoelastic foam [26], have been reported, but they cannot be easily used due to their high costs. In particular, overlays used in the surgeries are likely to become dirty or damaged by antiseptic solution. Therefore, this study attempted to present the standard by measuring the interface pressure of the cost-effective pressure-relieving overlay mostly used in South Korea.

This study has some limitations. First, since this study was conducted on healthy subjects with a wide age range, there may be a difference in the interface pressure of patients with intrinsic factors that may worsen the pressure injuries. Second, this study is limited by the large gender ratio. It is possible that the pressure was affected by the differences in musculoskeletal and fat distribution between adult males and females. Third, this study focused on only two locations (sacrum and heels) in only one position, but mechanical loads over tissues could change when body position changes and when different positions are used in surgery.

## 5. Conclusions

This study was conducted to access the interface pressure on the sacrum and both heels in the supine position as well as the subjective comfort of healthy volunteers on pressure-relieving overlays, such as PU foam, gel pads, and EC foam, in the operating table. As a result of the study, PU foam had the lowest interface pressures on the sacrum and both heels and was the highest in subjective comfort. Since this study measured the pressure in the conscious state in the supine position, we propose a study comparing the pressures in the unconscious state in various positions. Furthermore, since the interface pressure was measured on the sacrum and both heels in only a supine position, further studies are needed to make measurements in various positions.

## Figures and Tables

**Table 1:** General Characteristics of Subjects (*N* = 30).

Variables	Category	*n* (%) or Mean ± SD
Gender	Male	2 (6.5)
	Female	29 (93.5)
Age (years)		38.42 ± 9.64
Height (cm)		161.06 ± 5.92
Weight (kg)		57.32 ± 11.58
Body mass index		22.01 ± 3.85
	Underweight	4 (12.9)
	Normal	20 (64.5)
	Overweight	3 (9.7)

**Table 2:** Comparison of the average interface pressures of pressure-relieving overlays (mmHg).

Variables	Standard Mattress (*n* = 30)	PU ^1^ Foam (*n* = 30)	Gel Pad (*n* = 30)	EC ^2^ Foam (*n* = 30)	F	*p*	Post-Hoc
	Mean ± SD						
Sacrum	56.10 ± 23.03 ^a^	31.55 ± 13.60 ^b^	57.42 ± 35.61 ^c^	47.03 ± 32.29	5.82	0.001	a,c > b
Both heels	84.68 ± 36.47 ^a^	41.39 ± 13.29 ^b^	63.58 ± 32.31 ^c^	48.10 ± 27.01 ^d^	13.97	<0.001	a > b,c,d; c > b

^1^ PU = polyurethane. ^2^ EC = egg crate.

**Table 3:** Comparison of the subjective comfort of pressure-relieving overlays.

Variable	Standard Mattress (*n* = 30)	PU ^1^ Foam (*n* = 30)	Gel Pad (*n* = 30)	EC ^2^ Foam (*n* = 30)	F	*p*	Post-Hoc
	Mean ± SD						
Subjective comfort ratings	5.38 ± 1.76 ^a^	7.00 ± 2.09 ^b^	5.91 ± 1.74 ^c^	6.57 ± 2.01 ^d^	13.46	<0.001	a < b,d; d > c

^1^ PU = polyurethane. ^2^ EC = egg crate.
